# Social stress, cortisol awakening response and sex: association with hippocampus and amygdala volume

**DOI:** 10.1186/s13293-025-00801-9

**Published:** 2025-12-09

**Authors:** Rui Wang, Hannes Noack, Leandra Kuhn, Vanessa Nieratschker, Ute Habel, Birgit Derntl, Lydia Kogler

**Affiliations:** 1 Department of Psychiatry and Psychotherapy, Tübingen Center for Mental Health (TüCMH), Medical Faculty, Women’s Mental Health & Brain Function, University of Tübingen, Calwerstraße 14, 72076 Tübingen, Germany; 2 Department of Psychiatry, Psychotherapy and Psychosomatics, RWTH Aachen University, Aachen, Germany; 3 Department of Psychiatry and Psychotherapy, Molecular Psychiatry, Tübingen Center for Mental Health (TüCMH), Medical Faculty, University of Tübingen, Tübingen, Germany; 4 Institute of Neuroscience and Medicine (INM-10), Research Centre Jülich, Jülich, Germany; 5 German Center for Mental Health (DZPG), Partner site Tübingen, 72076 Tübingen, Germany; 6 LEAD Graduate School and Research Network, University of Tübingen, 72074 Tübingen, Germany

**Keywords:** Hippocampus, Amygdala, Gray matter volume, Sex difference, Cortisol awakening response, Social stress

## Abstract

**Objective:**

Volumes of the hippocampus and amygdala, both major hubs for neural stress regulation amongst others, are associated with social stressors, cortisol awakening response (CAR) and sex. Importantly, the interplay of these different factors in affecting the morphology of both brain regions remains unclear. This study aimed to elucidate the intricate influence of these factors on grey matter volumes (GMV) of the hippocampus and amygdala.

**Methods:**

We analyzed associations between structural brain data, self-reported chronic social stress (including subscales on social tension, social overload, lack of social recognition and social isolation) and CAR of 83 healthy participants (40 females) with multiple regression analyses.

**Results:**

In males, but not females, higher levels of social tension were associated with lower bilateral hippocampal GMV. Amygdala GMV was related to CAR and social stress, with social overload being associated with reduced amygdala GMV in individuals not showing the typical CAR (reflecting a blunted physiological response to awakening), while the opposite pattern emerged in those with a typical CAR.

**Conclusions:**

The association between chronic social stress and HC and AMY volume is interacting with CAR-pattern and sex. The brain morphology in males and in individuals with an atypical CAR showed reductions in association with chronic social stress. Our findings point to a complex interaction between social stress, cortisol patterns, sex and brain architecture, which needs to be assessed in more detail in future research.

**Supplementary Information:**

The online version contains supplementary material available at 10.1186/s13293-025-00801-9.

## Abstract (plain language summary)

The hippocampus and the amygdala are two important brain regions that help us cope with stress. How everyday social stress, biological stress responses, and sex work together to influence these brain regions is still not well understood.

In our study, we investigated 83 healthy adults, including both women and men. We measured how much social stress people reported in their daily lives, such as feeling tense in social situations, feeling overwhelmed by social demands, or lacking social recognition and support. We also measured the “cortisol awakening response,” which is the typical rise in the stress hormone cortisol that happens shortly after waking up in the morning. We then examined how these factors are related to the size of the hippocampus and amygdala using brain scans.

We found that men who reported higher levels of social tension had smaller hippocampus volumes, while this was not observed in women. People whose cortisol did not rise after awakening - a pattern indicated an “atypical cortisol awakening response” - showed smaller amygdala volumes when they experienced too many social responsibilities and interactions. Those subjects with a typical cortisol pattern after awakening showed the opposite effect. These results suggest that in men and in individuals with an atypical cortisol response the negative association between chronic social stress and brain structures is stronger.

**Supplementary Information:**

The online version contains supplementary material available at 10.1186/s13293-025-00801-9.

## Highlights

Social stress-related brain changes are associated with sex and HPA axis.

Social tension is linked to reduced hippocampus volume in males but not in females.

Social overload is associated with reduced amygdala in subjects with a lower CAR.

Maladaptive HPA axis function may increase the negative effects of social stress.

**Supplementary Information:**

The online version contains supplementary material available at 10.1186/s13293-025-00801-9.

## Introduction

Stress reactivity involves a distributed neural circuitry, with key brain regions including the hippocampus, amygdala, and prefrontal cortex playing central roles in adaptive responses to stressful situations [44, 45, 52]. However, when stress is prolonged, such as in chronic social stress, these regions, particularly the hippocampus and the amygdala, are adversely affected [18, 45]. Structural reductions in these areas have been observed in rats exposed to repeated social defeats [54], as well as in humans subjected to early-life stress [23] and stress within the past months [55, 73, 83]. Furthermore, reductions in their volumes are linked to stress-associated disorders, such as depression [53] and post-traumatic stress disorder [16]. These findings emphasize the importance of the hippocampus and the amygdala in stress responses and indicate their vulnerability to chronic stress exposure.

Both brain regions are significantly influenced by glucocorticoids (primarily cortisol in humans), due to their high density of gluco- and mineralocorticoid receptors [15, 75]. Cortisol regulates the stress response by binding to these receptors, a process closely linked to the hypothalamus-pituitary-adrenal (HPA) axis, which controls cortisol secretion through a cascade of responses [27]. In healthy individuals, cortisol exhibits a distinct circadian rhythm, characterized by a significant increase in cortisol levels around 30–45 min after awakening in the morning, the so-called cortisol awakening response (CAR), which is then followed by a subsequent decline over the day [58]. Chronic stress is known to disrupt this pattern, leading to a reduced increase in cortisol after awakening [17], possibly due to prolonged activation of the HPA axis, which impairs the ability to respond adaptively after awakening. This atypical CAR pattern is thought to reflect impaired neuroendocrine function and a dysregulated circadian rhythm [71], which is further related to brain atrophy and reduced volumes of the hippocampus [59].

Research reports sex differences in reaction to social stress. Females show, e.g., greater response to social rejection [72] and are more susceptible to exhaustion from social media overload compared to males [81]. Furthermore, a review by Gray et al. [22] assessing the associations between glucocorticoids and sex hormones such as estradiol or testosterone with stress-induced brain changes further emphasizes the importance of considering sex as an impacting factor on stress-related brain regions. Evidence from animal studies demonstrates consistent results: In males, chronic stress, induced by chronic restraint task, causes dendritic atrophy in the hippocampus and the amygdala, whereas females do not show the same pattern [19, 40], unless they are ovariectomized (i.e., significantly decreased estradiol levels) [47, 48]. This suggests that sex hormones may attenuate the effects of chronic stress on hippocampus dendritic atrophy [14, 46]. In humans, sex-dependent associations between stress-induced cortisol levels and striatal-limbic structures were reported, with positive associations observed in males and negative ones in females [26]. Additionally, females were observed to have greater functional connectivity within the amygdala network in response to social exclusion compared to males [10]; and males showed more pronounced neural activation in the inferior frontal gyrus and insula [36]. In stress-related mental disorders, sex differences appeared with an atypical, reduced CAR going along with smaller left hippocampus volumes in male patients with first-episode psychosis and smaller bilateral hippocampus volumes in high-risk individuals, whereas this association was not seen in females [59, 60]. So far, it remains unclear whether similar sex-specific patterns between CAR and volumes of stress-regulating brain areas such as the hippocampus and the amygdala appear in healthy individuals as well.

Taken together, the interplay between chronic social stress and CAR on hippocampus and amygdala volumes, as well as the effect of sex on the relationship between chronic social stress, CAR and these brain structures remain unclear. Thus, the current study aims to explore interactions between chronic social stress, CAR, and sex affecting grey matter volume (GMV) of the hippocampus and the amygdala. More specifically, we exploratively assessed whether the following variables were associated with the GMV of the hippocampus and amygdala: (1) the interaction between chronic social stress and CAR; (2) the interaction of chronic social stress and sex; and (3) the interaction of sex and CAR. Our hypotheses are as follows: (1) individuals with lower CAR experiencing high chronic social stress will show reduced GMV of the hippocampus and the amygdala, potentially due to their deficient HPA axis regulation [71]; (2) in males, but not in females, higher chronic social stress will be associated with lower GMV in the hippocampus and amygdala [19, 40]; and (3) in males, but not in females, a lower CAR will be associated with reduced hippocampus volumes [59, 60].

## Methods

### Sample

Participants were part of a multi-center study [35, 36], recruited through public advertisements at the University of Tuebingen and the RWTH Aachen University, Germany, and were between 18 and 35 years of age. Eligibility for the study was evaluated through a semi-structured interview and the screening version of the German Structured Clinical Interview for Diagnostic and Statistical Manual of Mental Disorders (SCID, Wittchen et al. [79]. Inclusion criteria were right-handedness, nonsmokers, normal weight (17 < BMI < 30), a regular day-night cycle (no shift working, jet lag). Exclusion criteria included any self-reported history of neurological or mental disorders, pregnancy, as well as common requirements for MRI scanning, such as no metal parts in or on the body, tattoos, pacemakers, etc. Additionally, only females using oral contraceptives were included during their pill-intake phase to minimize the impact of hormonal fluctuations on cortisol reactivity [32]. Participants completed the State-Trait Anxiety Inventory (STAI, Spielberger et al. [70] and the Beck Depression Inventory-II (BDI, Beck [5, 30], to assess their anxiety and depression levels.

Of note, the term “sex” used throughout this paper refers to the biological sex assigned at birth, as reported by participants, and is categorized as female or male in accordance with the Sex and Gender Equity in Research (SAGER) guidelines [25].

The study protocol was approved by the ethics committees of the medical faculties at the RWTH Aachen University (EK 212/16) and the University of Tuebingen (409/2015BO2), and was conducted in accordance with the declaration of Helsinki [80]. This work is published as a preprint on bioRxiv (DOI: 10.1101/2025.03.20.644300).

### Cortisol collection

Saliva samples were collected on a normal working-day with a typical daily routine using Cortisol Salivettes (Sarstedt, Nürnbrecht, Germany) immediately after awakening, 15, 30, 45, and 60 min post-awakening. Participants received detailed instructions during the screening appointment a few days before the MR session and returned the samples when they came for the MR session. Participants placed a synthetic fiber swab in their mouth for one minute to absorb saliva and were told not to chew the swab. Participants recorded the exact time of each sample collection to ensure adherence to the timing protocol. They were instructed not to brush their teeth, drink, eat or smoke prior to the sampling procedures, to abstain from alcohol and nicotine consumption and excessive exercise on the day before sample collection, and to maintain their usual waking times and daily routines on the sampling day. After collection, participants stored the samples in a fridge and brought them to the laboratory in the afternoon of the same day, when the MR-scanning and the assessment of chronic social stress took place. The samples were kept in the refrigerator or freezer until they were sent for analysis to the Institute of Pharmaceutical Sciences, University of Tuebingen.

Salivary cortisol was measured using a liquid chromatography (LC)-mass spectrometry assay. Samples preparation involved: thawing at 4 °C, protein precipitation, solid-phase extraction using Oasis PRIME hydrophilic-lipophilic balance material on a 96-well plate, drying under nitrogen, and reconstituting in 50 µL of a methanol-water solution (30:70; v/v) in a sealed 96-well collection plate. Chromatographic separation was performed using a MicroLC 200 Plus instrument (Sciex, Framingham, MA, USA), with analyte detection carried out on a QTRAP 4500 triple-quadrupole mass-spectrometer (Sciex) in negative ionization mode. For accurate quantification, a surrogate calibrant method using Cortisol-d4 in a true saliva matrix was employed. The method demonstrated a linear range for cortisol quantification from 0.062 to 75.5 ng/mL and was validated in accordance with FDA guidelines.

### Chronic social stress

To assess chronic social stress levels, we investigated subscales of the Trier Inventory for the Assessment of Chronic Stress (TICS, Schulz et al. [66, 67], assessing stress resulting from high social demands or a lack of social need satisfaction appearing within the previous 3-months: “social overload” (the feeling of being overwhelmed due to the number and intensity of social interactions and responsibilities); “lack of social recognition” (not receiving sufficient appreciation or recognition from others in social situations); “social tension” (conflicts and tense interactions in social networks); and “social isolation” (feelings of loneliness and isolation from others).

### MRI data acquisition and pre-processing

For structural magnetic resonance imaging (MRI) data of the brain, participants were scanned with a T1-weighted magnetization prepared rapid acquisition gradient-echo (MPRAGE) sequence. The acquisition parameters were as follows: TR = 5,000 ms, TE = 2.98 ms, flip angle = 9◦, FOV = 256 × 256 mm, 176 slices, voxel size = 1 mm^3^, interleaved, with a distance factor of 50%.

The preprocessing of T1-weighted images was performed by using the Computational Anatomy Toolbox (CAT12, https://neuro-jena.github.io/cat/), the Statistical Parametric Mapping software (SPM12, https://www.fil.ion.ucl.ac.uk/spm/) and MATLAB R2023a (Mathworks, Natick, MA, USA, https://uk.mathworks.com/). The cross-sectional data segmentation tool was run with the default settings provided by CAT12. Each participant’s T1-weighted image underwent spatial normalization and segmentation into grey matter (GM), white matter (WM) and cerebrospinal fluid (CSF). These images were normalized to a standard stereotactic space (MNI template), with a resulting voxel size of 2 mm for the normalized images. After preprocessing, the modulated and normalized GMV images for each participant were smoothed by an 8 mm full width at half maximum (FWHM) isotropic Gaussian kernel.

**Fig. 1:**
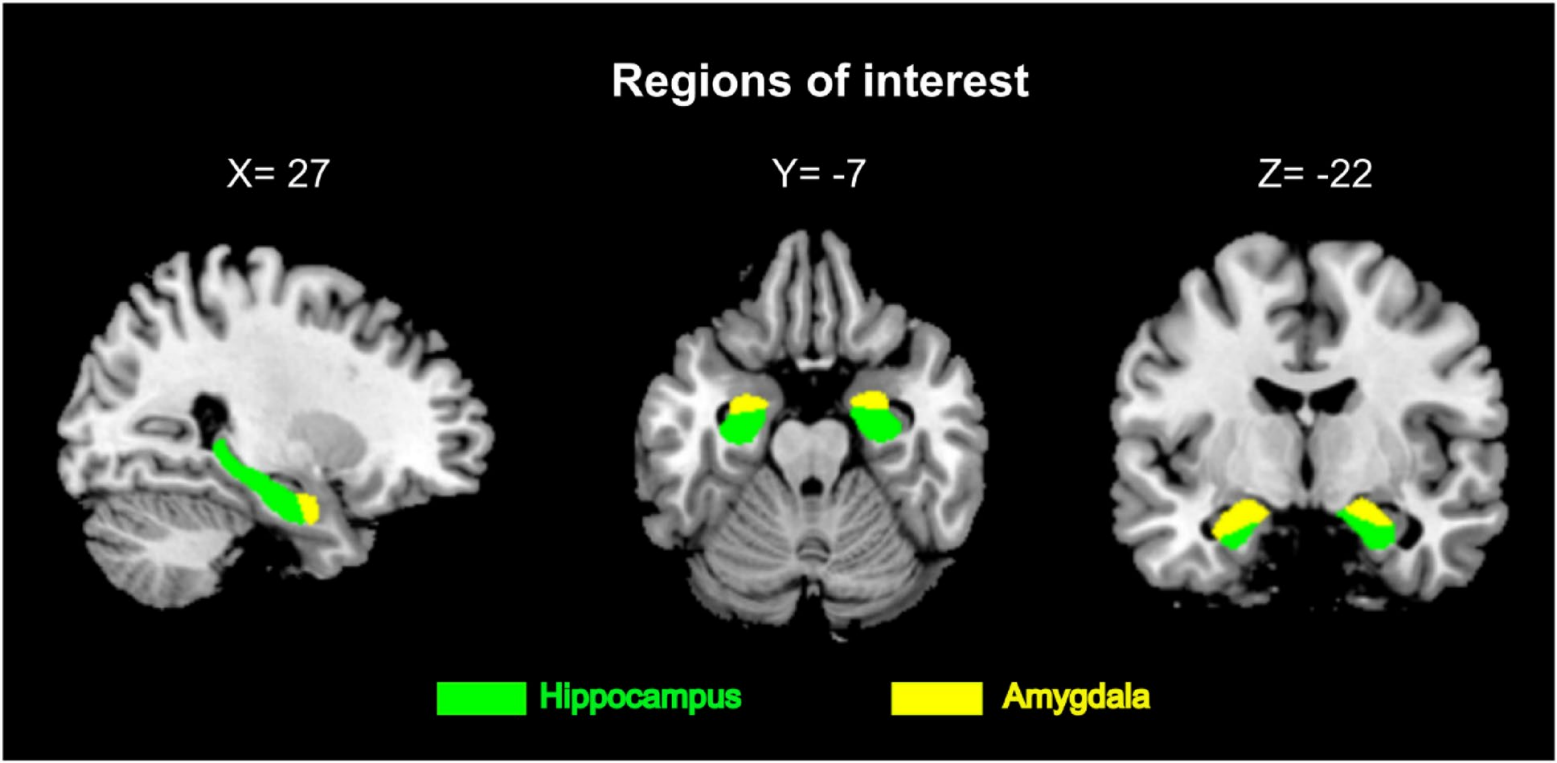
Regions of interest in MNI space at coordinate X = 27, Y = −7, Z = −22

For region of interest (ROI) analyses, masks were created based on the neuromorphometrics atlas using the Image Calculator tool in SPM12. The location of the ROIs in this study is shown in Fig. 1. GMVs were extracted from the left and right hippocampus, and the left and right amygdala by “get_totals script, G. Ridgeway, http://www0.cs.ucl.ac.uk/staff/gridgway/vbm/get_totals.m, date of access: June 27, 2023”, as done in a previous study [56].

### Statistical analyses

Data were analyzed using IBM SPSS Statistics 29 (https://www.ibm.com/de-de/products/spss-statistics). Based on established criteria, participants were categorized into responders and non-responders according to their cortisol increase at 30 min after awakening. Responders showed a cortisol increase of more than 50% from baseline to 30 min post-awakening, while non-responders showed an increase of less than 50% [13, 82].

Multiple regression analyses were performed to examine whether chronic social stress, CAR and sex are predictive of GMV. Separate analyses were conducted for the left and right hippocampus and amygdala as dependent variables. For each side of each ROI, four different models were applied corresponding to four subscales of chronic social stress. The predictors in the regression model were sex (females/males), CAR (responders/non-responders), chronic social stress level, and their interactions (chronic social stress × sex; chronic social stress × CAR; sex × CAR). Total intracranial volume (TIV) and age were included as covariates of no interest in each model. In cases where significant interactions were identified, partial correlation analyses were performed, controlling for TIV and age, to clarify the associations within each group. To reduce multicollinearity, chronic social stress scores were mean-centered. The CAR group and sex were dummy-coded. To control for multiple comparisons, p-values were adjusted using false-discovery-rate (FDR).

## Results

### Sample characteristics

A total of 83 participants (40 females) were included in the current study based on the data availability within the original database. Of these, 45 participants (22 females) were classified as responders and 38 participants (18 females) as non-responders according to the increasing levels of cortisol after awakening. Consequently, responders showed higher area under the curve with respect to the increase (AUCi) in the CAR compared to non-responders (t(81) = 3.63, *p* < 0.001), aligned with the classification criteria. No other significant differences were shown between groups. Moreover, results of the chi-square test indicated that there was no significant difference in the distribution of CAR responders and non-responders between females and males (χ² = 0.019, *p* = 0.99). Additionally, males were older compared to females (t(81) = 2.14, *p* = 0.035), and as expected and in accordance with the literature, males exhibited a larger TIV than females (t(81) = 8.653, *p* < 0.001). No further significant sex differences in sample characteristics were observed. Please see Table 1 for details.

The expert consensus guidelines on the CAR by Stalder et al. [71] indicate that delay between awakening and initiation of sampling is common in CAR studies. In our study, we observed a slight delay in the CAR sampling, with an average delay of 1.83 min (SD = 4.75), which is notably shorter than the average delay (7.1–24.8 min) reported by Stalder et al. [71]. Moreover, no significant differences in sampling time delays were found between females and males (*p* = 0.78), nor between responders and non-responders (*p* = 0.14).

**Table 1:** Sample characteristics

	Overall (*N* = 83)	Females (*N* = 40)	Males (*N* = 43)	*P* ^1^	Responders (*N* = 45)	Non-responders (*N* = 38)	*P* ^2^
Age (years)	23.22 ± 3.11	22.48 ± 2.56	23.91 ± 3.43	0.035	22.64 ± 2.98	23.89 ± 3.16	0.068
BDI-II	2.99 ± 3.34	2.83 ± 3.45	3.14 ± 3.26	0.671	3.04 ± 3.46	2.92 ± 3.22	0.868
STAI-State	43.41 ± 7.03	44.24 ± 7.50	42.66 ± 6.57	0.323	42.45 ± 7.08	44.53 ± 6.89	0.195
STAI-Trait	33.61 ± 7.78	33.73 ± 7.26	33.50 ± 8.34	0.897	33.16 ± 7.11	34.13 ± 8.56	0.576
Cortisol after awakening (ng/mL)							
CAR - AUCg	475.96 ± 655.17	517.64 ± 754.39	438.17 ± 556.65	0.679	450.69 ± 592.72	506.70 ± 731.20	0.703
CAR - AUCi	114.99 ± 304.86	117.08 ± 243.41	113.08 ± 354.43	0.367	218.32 ± 345.52	−10.69 ± 183.34	< 0.001
Awakening time (a.m.)	7:27 ± 1:11	7:16 ± 1:13	7:41 ± 1:07	0.190	7:15 ± 1:05	7.43 ± 1:12	0.051
Sleep duration (hours)	7.68 ± 1.22	7.87 ± 1.24	7.45 ± 1.17	0.132	7.60 ± 1.13	7.57 ± 1.23	0.917
Chronic social stress							
Social overload	7.00 ± 4.77	6.63 ± 4.42	7.35 ± 5.10	0.493	6.36 ± 4.29	7.76 ± 5.24	0.182
Lack of social recognition	4.01 ± 2.72	3.98 ± 2.65	4.05 ± 2.81	0.905	3.67 ± 2.59	4.42 ± 2.83	0.209
Social tension	5.25 ± 4.66	5.00 ± 4.82	5.49 ± 4.54	0.636	4.89 ± 4.42	5.68 ± 4.94	0.442
Social isolation	6.36 ± 4.23	5.88 ± 3.76	6.81 ± 4.63	0.316	6.31 ± 4.42	6.42 ± 4.06	0.907
TIV	1563.55 ± 158.48	1450.36 ± 116.78	1668.85 ± 113.22	< 0.001	1569.80 ± 165.93	1556.16 ± 151.04	0.699

Values are presented as mean ± standard deviation. Sleep data were from 56 participants due to missing sleep data for some participants. ^1^ indicates p-value for the differences between females vs. males. ^2^ indicates p-value for the differences between responders vs. non-responders. BDI-II = Beck Depression Inventory-II; STAI = State-Trait Anxiety Inventory; CAR = cortisol awakening response; AUCg = area under the curve with respect to the ground; AUCi = area under the curve with respect to the increase

### Associations between chronic social stress, CAR and sex with GMV of ROIs

**Fig. 2:**
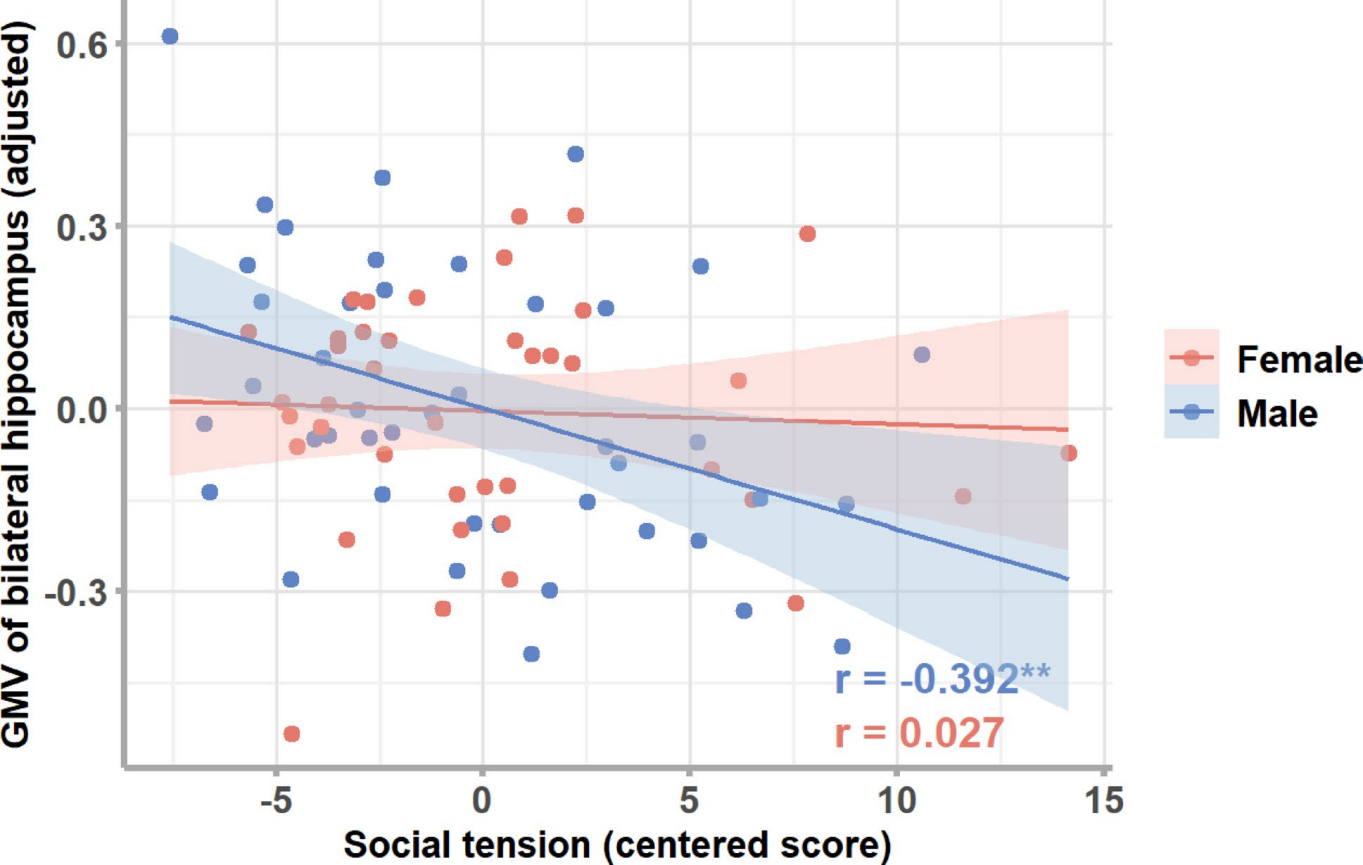
Associations between social tension and GMV of the bilateral hippocampus in females and males

*Hippocampus* For bilateral hippocampus the model including values from the subscale social tension was significant (R^2^ = 0.544, *p* < 0.001; see Table S1 in the supplementary material) showing a significant interaction of social tension × sex (β = 0.28, p_uncorr_ = 0.023, p_FDR_ = 0.046; see Fig. 2). Both left and right hippocampi showed the same pattern which we report in the supplementary material (see Figure S1 and Table S5). To disentangle the interactions, post-hoc partial correlation analyses revealed a significant negative correlation between social tension and hippocampus GMV in males (*r* = −0.392, p_uncorr_ = 0.011, p_FDR_ = 0.022), whereas no significant correlation was observed in females (*r* = 0.027, p_uncorr_ = 0.873, p_FDR_ = 0.873).

No other predictor variables or their interactions were significant for bilateral hippocampus GMV at the corrected level (see Table S1-S4 for statistical parameters on all regression models; Table S5-S8 and Figure S1 for the regression models on left and right hippocampus separately).

**Fig. 3:**
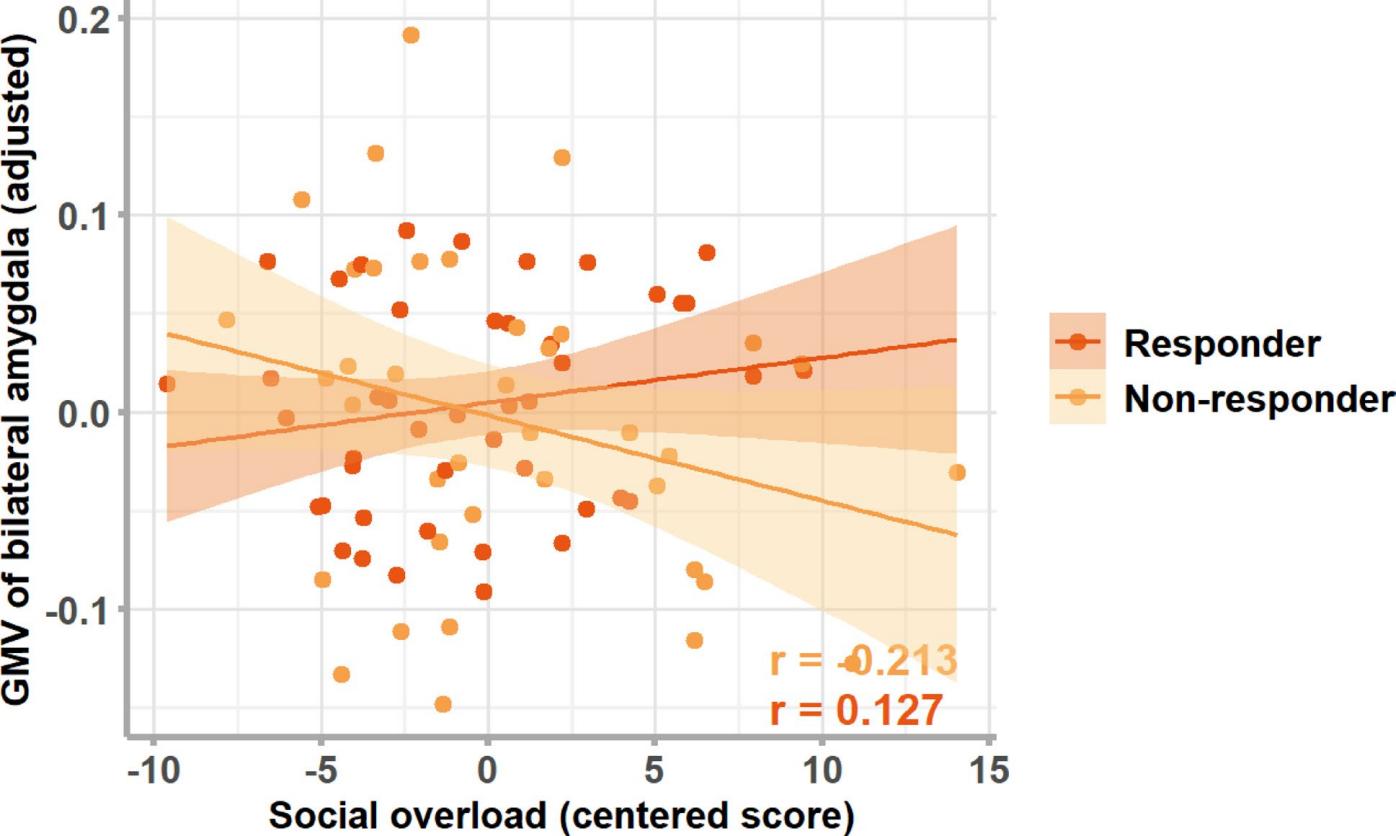
Associations between social overload and GMV of the bilateral amygdala in responders and non-responders. CAR = cortisol awakening response

*Amygdala* For the bilateral amygdala, the model including values from the subscale social overload was significant (R^2^ = 0.573, *p* < 0.001; see Table S2) showing a significant interaction between social overload × CAR (β = −0.27, p_uncorr_ = 0.017, p_FDR_ =0.034; see Fig. 3). Post-hoc partial correlations show a positive, although not significant, correlation in responders (*r* = 0.127, p_uncorr_ = 0.417) and a negative, although not significant, correlation in non-responders (*r* = −0.213, p_uncorr_ = 0.212). No other predictor or interactions showed a significant association with bilateral amygdala GMV (see Table S1-S4 for statistical parameters for all regression models; and Table S5-S8 and Figure S2 for the regression models on left and right amygdala separately).

## Discussion

The current exploratory study examined the associations between different facets of chronic social stress, the CAR, sex, and the volume of the hippocampus and amygdala in healthy young individuals. Our results show that higher amounts of self-reported socially tense, conflictual interactions are linked to reduced bilateral hippocampus GMV in males, whereas this pattern differs in females. Furthermore, CAR non-responders who are overwhelmed by the number of social responsibilities (social overload) show reduced amygdala GMV, whereas CAR responders show the opposite pattern.

### The relationship between sex and social stress

Females and males differ in the way social interactions are associated with hippocampus volume, with males who reported higher socially tense interactions showing decreased hippocampus volume. Previous studies with mixed samples of females and males, have consistently shown that high levels of perceived life and social stress are associated with reduced hippocampus volume (e.g., [23, 41, 55, 83], and a history of emotional abuse is associated with reduced hippocampus volume in males but not in females [65]. This sex-specific pattern aligns well with results reported in animal studies: In male rats, chronic stress was also found to cause dendritic atrophy of hippocampus pyramidal neurons, whereas this was not seen in females [19, 46]). Recent human reviews have highlighted that the hippocampus is particularly susceptible to stress-related sex differences [20]. In general, prolonged exposure to stress prevents relaxation and the maintenance of homeostasis, which subsequently can alter brain structure by changing neuronal morphology and by suppressing neurogenesis [31, 50]. Our results taken together with these reports in the literature point to sex-specific neuro-morphological changes.

One line of argumentation takes sex hormones such as estradiol and progesterone and their neuroprotective effects into account. Estradiol can enhance dendritic complexity and synaptic spine density in the hippocampus, thereby improving brain volume recovery from injury [21, 28, 38]. Progesterone further augments the effect of estradiol [21]. Both estradiol and progesterone levels are associated with stress reactions in females although not only in a protective way [3, 6, 32]. Thus, protective effects of sex hormones against the dendritic retraction in the hippocampus caused by chronic stress [40, 46, 48], might explain the lack of hippocampus volume reduction in socially stressed females.

Additionally, all females in this study were using OC. While controlling for the potential effects of fluctuations in estradiol and progesterone, it is worth mentioning that most OC contain synthetic estradiol such as ethinylestradiol and/or a synthetic progesterone such as levonorgestrel. Ethinylestradiol, similar to natural sex hormones, induces the synthesis of corticosteroid-binding globulin (CBG) [49, 78], leading to elevated CBG levels during OC use. Since CBG binds to circulating cortisol, the amount of free and bio-active cortisol available to bind to glucocorticoid receptors in the hippocampus is reduced [19, 42], potentially mitigating the negative effects of unbound cortisol on hippocampus volume. Furthermore, OC use has been shown to influence brain structures. Previous studies consistently report a positive association between OC use and total GMV, as well as an increase in volumes in regions such as the hippocampus, parahippocampus gyrus, and fusiform gyrus, which is also related to the duration and type of OC use [8, 56, 57].

Overall, biological protective mechanisms in healthy females appear to prevent negative effects of social stress on hippocampus morphology. This seems to contrast with previous arguments that females are more susceptible to social stress, evidenced by higher cortisol responses to social rejection [72], and that they are more likely to be diagnosed with some stress-related mental disorders [39, 43]. Our results indicate that they may show less pronounced volume reductions in the hippocampus following chronic social stress, while males show a higher reduction. Interestingly, social stress such as crowded housing does impact male rats more than females [9]. It is additionally noteworthy that psychosocial stress, which besides a social evaluation and social comparison as stressors also includes achievement as a stressor, improves social processing (social cognition, empathy) in males, but seem to show no or an opposite effect in females [51, 69]. Thus, it might be that chronic social stress induces increased empathic stress in males, which potentially is also associated with cerebral changes in the hippocampus. Our results on social tension highlight the importance of considering sex-specific and neuroendocrine factors in stress-induced brain remodeling.

### The association between CAR and social stress

Our data further revealed an interaction of chronic social stress and CAR on brain volume. We observed distinct patterns for CAR non-responders and CAR responders, with a negative association in non-responders and a positive association in responders. This suggest that lacking the typical CAR pattern is linked to a stronger association between being overloaded with social interactions and responsibilities and a reduced amygdala volume. An absent cortisol response to awakening reflects an altered neuroendocrine system and an impaired HPA axis regulation, which is crucial for the maintenance of homeostasis as part of the neurobiological circadian recovery system [71]. The amygdala, a key structure involved in regulating emotions, anxiety, and fear responses [61, 68], is particularly sensitive to psychosocial stress [63]. Its functional network is associated with basal cortisol levels [33]. Our data now indicates that amygdala GMV shows distinct patterns in CAR responders vs. non-responders in association with social overload. Non-responders compared to responders showed a negative association, with more social overload being associated with reduced amygdala volumes. Interestingly, also in social media use, an association with amygdala GMV was reported: An excessive social media use is associated with reduced bilateral amygdala GMV [24]. Additionally, patients with damaged amygdala volume seem to have a smaller social network size [4], and in depressed patient, amygdala volumes are negatively correlated with CAR [64]. Thus, a maladaptive stress response and recovery system may amplify the negative effects of having too much social responsibilities on brain structure. In contrast, a functional CAR indicates a neuroprotective and adaptive HPA axis, enabling the individual to effectively adapt to external social stressors and thereby probably mitigate the adverse effects of chronic stress on amygdala volume. Thereby, in responders, the effective management of the circadian HPA axis for restoration and recovery is linked to a positive association between social interactions and amygdala volume. Non-responders, however, may lack this protective mechanism and flexibility to adapt to external social stressors, which is preventing the body’s homeostasis. This seems further to be associated with reduced amygdala volumes when being chronically exposed to too much social responsibilities. A healthy cortisol circadian rhythm to awakening – indicated by a normal CAR pattern – may promote adaptive regulation of the HPA axis in response to chronic socially stressful situations. Our study adds that the association between social stress and amygdala volume further is associated with the adaptive neurobiological regulation mechanisms of the individual. There seems to be an association between protective effects of a well-functioning circadian neurobiological recovery system, social overload and amygdala volume. Nevertheless, our data so far is only exploratory and correlational, and a causal relationship between the variables needs to be investigated using longitudinal or interventional designs.

Furthermore, in the current study no significant interactions with CAR, sex and hippocampus or amygdala volume were found for chronic social isolation and lack of social recognition. The lack of associations may be related to their characteristics, reflecting prolonged social neglect and insult compared to acute conflicts that strongly activate physiological stress responses [74]. Therefore, our findings suggest that a lot of social responsibilities is a prominent stressor that is associated with amygdala volume when the neurobiological recovery system is missing.

A dysregulation of the HPA axis is commonly reported in mental disorders [11, 34, 76]. Specifically for the CAR, previous studies indicate that depression severity is associated with blunted CAR [2], while a higher waking cortisol level is related to an increased risk of depression [1]. A blunted CAR has also been observed in patients with post-traumatic stress disorder [12] and psychosis [7]. Furthermore, Weber et al. [77] found that patients with functional neurological disorder, a neuropsychiatric condition characterized by a variety of disabling neurological symptoms, exhibit a blunted CAR, and reduced amygdala volumes, which aligns with our findings. Thus, a blunted CAR, along with socially induced overload appears to be associated with mental health issues and structural brain changes. Our findings now add that the hippocampus and the amygdala are reshaped by chronic social stress even in healthy individuals in a sex-specific way and associated with the CAR pattern. The current study suggests that the typical CAR pattern indicates an adaptive neurobiological recovery system that may help to prevent negative effects of social stress and might also impact the development of stress-associated mental disorders.

### Future directions

Endogenous and exogenous sex hormones that are varying throughout the menstrual cycle, with hormonal contraceptive intake, and during the menopausal transition, impact CAR [29, 71]. The current study included only females using oral contraceptives which may result in a lower CAR [29]. The impact of endogenous and exogenous sex steroids in females and males at different hormonal stages on CAR and its association with brain structure needs to be addressed in future research [37]. Second, due to limited resources within the current project, the exact sampling time of CAR relied on self-reports only. CAR samples were collected on one day, which may not accurately reflect the usual, longitudinal CAR patterns. To improve the validity of collected samples, additional methods to verify sampling and awakening time would be desirable [62]. Third, while this study focused on structural changes in the hippocampus and the amygdala, future research should explore how the network of these brain regions and other regions involved in stress reaction and regulation are affected. This would provide a more comprehensive understanding of stress-related neurobiological mechanisms. Additionally, the different associations between chronic stress and brain structure observed in responders and non-responders in this study need to be further validated in patients with stress-associated mental disorders. To identify causal relationships, longitudinal and interventional studies would provide further insights into how sex, chronic social stress and CAR impact brain structure over time.

### Conclusion

The present study examined the association between effects of chronic social stress, CAR, sex and GMV in the hippocampus and the amygdala, highlighting the complex interplay of stress-related factors in these brain regions. Our findings demonstrate that chronic social tension is related to hippocampus volume differently in females and males, with a significant negative association between social tension levels and hippocampus GMV only in males. In females, higher levels of sex hormones might have a neuroprotective effect and potentially counteract the harmful effects of chronic social stress. In males, increased empathic stress might be associated with anatomical changes. Additionally, we observed that chronic social overload is related to amygdala volume differently in individuals who show a typical cortisol reaction to awakening and those who do not. A negative association between stress levels and GMV of amygdala was seen in non-responders. An inadequate cortisol response upon awakening may reflect underlying dysfunctions in the neurobiological, circadian recovery of the HPA axis, which further seems to be associated with prolonged social stress and brain structure. While a functional CAR indicates an adaptive reaction of the body to reallocate and restore the body’s homeostasis to internal and external influencing factors, a missing CAR, as seen in non-responders, indicates a dysfunction in this neurobiological circadian recovery system.

Overall, chronic social stress levels such as conflicts, socially tense and overwhelming interactions are negatively associated with the morphology of the hippocampus and the amygdala, particularly among males and CAR non-responders. Understanding the sex-specific relationship between stress and brain structure, as well as the association with adaptive function of the HPA axis, is essential for developing effective stress management and intervention strategies. These insights could help mitigate neural structural changes in individuals who are more vulnerable to social stress and could potentially prevent further development of stress-associated mental disorders.

## Supplementary Information

Supplementary Material 1

## Data Availability

The data are available upon reasonable request from the authors.
