# Supplementary material for: Social stress, cortisol awakening response and sex: association with hippocampus and amygdala volume
Source: Biol Sex Differ. 2025 Dec 9;16:104. doi: 10.1186/s13293-025-00801-9 (PMC12690870; doi:10.1186/s13293-025-00801-9)
Supplement: Supplementary file 1 — Supplementary Material 1 [file 13293_2025_801_MOESM1_ESM.pdf]

Analyses on bilateral hippocampus and amygdala

Table S1. Statistical parameters for the models on social tension, cortisol awakening response (CAR) and sex and grey matter volume (GMV) of the bilateral hippocampus and amygdala.

	**GMV of bilateral hippocampus**		**GMV of bilateral amygdala**	
	**β**	**t**	**β**	**t**
Age	0.03	0.38	0.09	1.13
TIV	0.59	**5.16***	0.58	**5.48***
Sex	-0.08	-0.59	-0.17	-1.32
CAR	-0.04	-0.36	-0.40	0.37
Social tension	-0.20	-1.55	-0.13	-1.07
Social tension × sex	0.28	**2.32****	0.19	1.66
Social tension × CAR	-0.26	**-2.14***	-0.14	-1.22
Sex × CAR	0.03	0.21	-0.02	-0.19
	$R^2 = 0.544$, $p < 0.001$		$R^2 = 0.565$, $p < 0.001$	

Note: TIV = total intracranial volume. * indicate significant at p < 0.05 uncorrected; ** indicate significant at p < 0.05 FDR-corrected.

Table S2. Statistical parameters for the models on social overload, CAR and sex and GMV of the bilateral hippocampus and amygdala.

	**GMV of bilateral hippocampus**		**GMV of bilateral amygdala**	
	**β**	**t**	**β**	**t**
Age	0.04	0.47	0.10	1.31
TIV	0.68	**5.62***	0.65	**6.25***

Sex	0.002	0.02	-0.10	-0.78
CAR	0.02	0.12	0.01	0.09
Social overload	0.05	0.36	0.11	0.91
Social overload × sex	0.08	0.71	0.06	0.61
Social overload × CAR	-0.22	-1.75	-0.27	**-2.44****
Sex × CAR	-0.04	-0.25	-0.07	-0.58
	R^2 = 0.431, p < 0.001		R^2 = 0.573, p < 0.001	

Note: TIV = total intracranial volume. * indicate significant at p < 0.05 uncorrected; ** indicate significant at p < 0.05 FDR-corrected.

Table S3. Statistical parameters for the models on lack of social recognition, CAR and sex and GMV of the bilateral hippocampus and amygdala.

	**GMV of bilateral hippocampus**		**GMV of bilateral amygdala**	
	**β**	**t**	**β**	**t**
Age	0.01	0.16	0.07	0.97
TIV	0.66	**5.42***	0.64	**6.08***
Sex	-0.03	-0.18	-0.13	-0.99
CAR	0.02	0.19	0.02	0.17
Lack of social recognition	-0.06	-0.39	-0.004	-0.03
Lack of social recognition × sex	0.13	1.13	0.10	0.99
Lack of social recognition × CAR	-0.14	-1.20	-0.19	-1.85
Sex × CAR	-0.03	-0.23	-0.07	-0.54
	R^2 = 0.482, p < 0.001		R^2 = 0.565, p < 0.001	

Note: TIV = total intracranial volume. * indicate significant at p < 0.05 uncorrected; ** indicate significant at p < 0.05 FDR-corrected.

Table S4. Statistical parameters for the models on social isolation, CAR and sex and GMV of the bilateral hippocampus and amygdala.

	GMV of bilateral hippocampus		GMV of bilateral amygdala	
	**β**	**t**	**β**	**t**
Age	0.03	0.34	0.09	1.16
TIV	0.65	**5.32***	0.63	**5.86***
Sex	-0.04	-0.24	1.14	-1.02
CAR	-0.03	-0.23	-0.03	-0.31
Social isolation	-0.007	-0.05	0.03	0.23
Social isolation × sex	-0.04	-0.32	-0.02	-0.17
Social isolation × CAR	0.08	0.72	0.07	0.72
Sex × CAR	0.01	0.09	-0.02	-0.16
	$R^2 = 0.406$, $p < 0.001$		$R^2 = 0.587$, $p < 0.001$	

Note: TIV = total intracranial volume. * indicate significant at $p < 0.05$ uncorrected; ** indicate significant at $p < 0.05$ FDR-corrected.

Analyses on left and right hippocampus and amygdala

Hippocampus: For both the left and right hippocampi, the models including values from the subscale social tension were significant (left: R^2 = 0.515, p < 0.001; right: R^2 = 0.542, p < 0.001; see Table S5 in the supplementary material) with each showing a significant interaction of social tension × sex (left: β = 0.294, p = 0.02; right: β = 0.260, p = 0.035; see Figure S1A-B). Post-hoc partial correlation analyses showed a negative correlation between social tension and hippocampus GMV for non-responders (left: r = -0.406, p = 0.014; right: r = -0.415, p = 0.012), while the correlation was not significant in responders (left: r = -0.052, p = 0.740; right: r = -0.076, p = 0.628).

Additionally, for both the left and right hippocampi, the interaction of social tension × CAR was significant (left: β = -0.265, p = 0.034; right: β = -0.237, p = 0.049; Figure S1C-D). Post-hoc partial correlation analyses showed a negative correlation between social tension and hippocampus GMV for non-responders (left: r = -0.406, p = 0.014; right: r = -0.415, p = 0.012), while the correlation was not significant in responders (left: r = -0.052, p = 0.740; right: r = -0.076, p = 0.628).

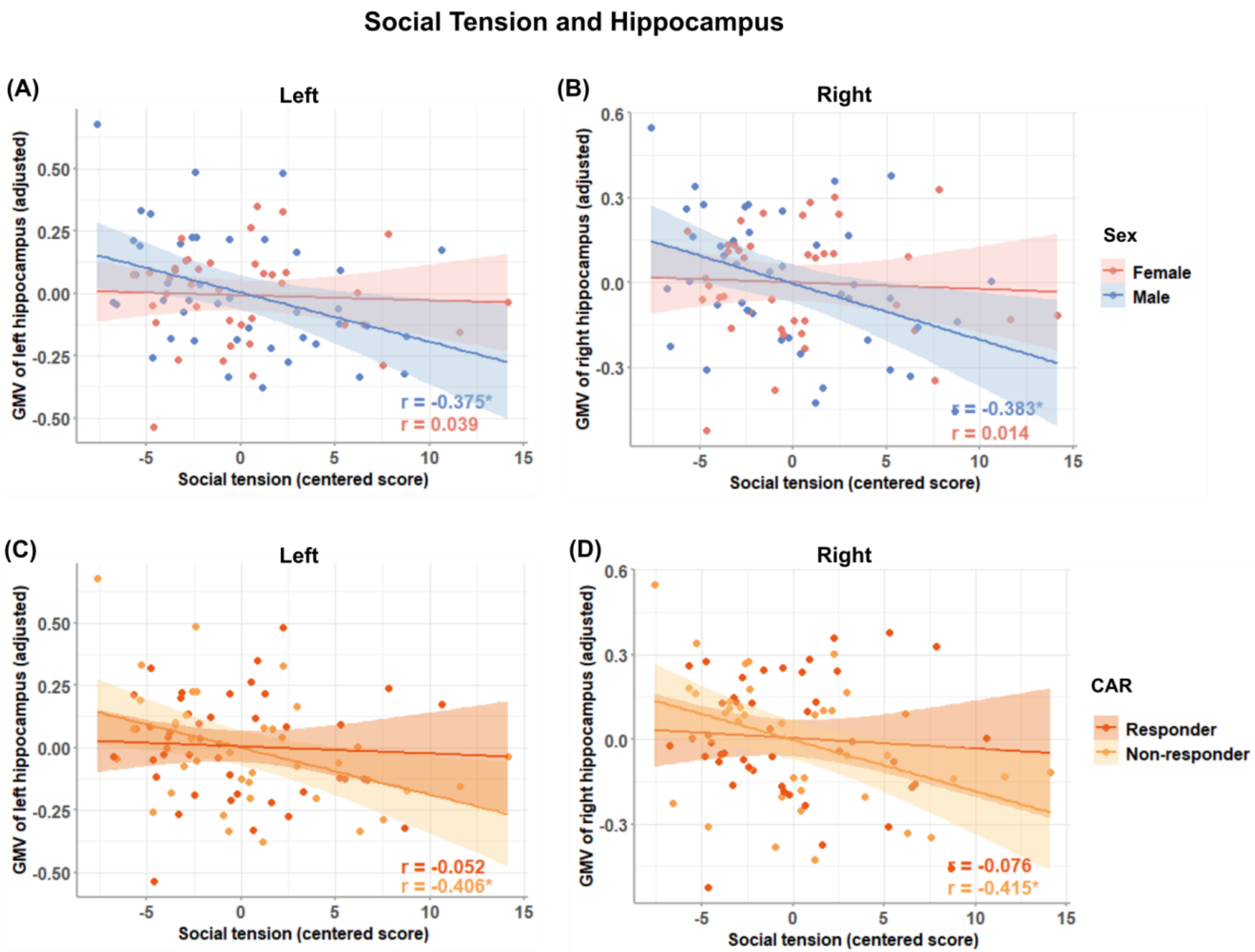

Figure S1. Disentangling the significant interactions of social tension x sex (A-B) and social tension x cortisol awakening response (CAR) (C-D). Males show a significant negative correlation between the amount of socially tense interactions and the hippocampus volume, whereas females show a positive association (left hippocampus: A, right hippocampus: B). Non-responders show a significant negative correlation between socially

tense interactions and the hippocampus volume, whereas responders show a positive association (left hippocampus: C; right hippocampus: D).

Amygdala: For both the left and right amygdalae, the models including data from the subscale social overload were significant (left: $R^2 = 0.582$, $p < 0.001$; right: $R^2 = 0.609$, $p < 0.001$; see Table S6) with each showing a significant interaction between social overload × CAR (left: β = -0.252, p = 0.032; right: β = -0.279, p = 0.015; Figure S2A-B). Post-hoc partial correlations were not significant for responders (left: r = 0.191, p = 0.220; right: r = 0.192, p = 0.218) and non-responders (left: r = -0.225, p = 0.113; right: r = -0.275, p = 0.105).

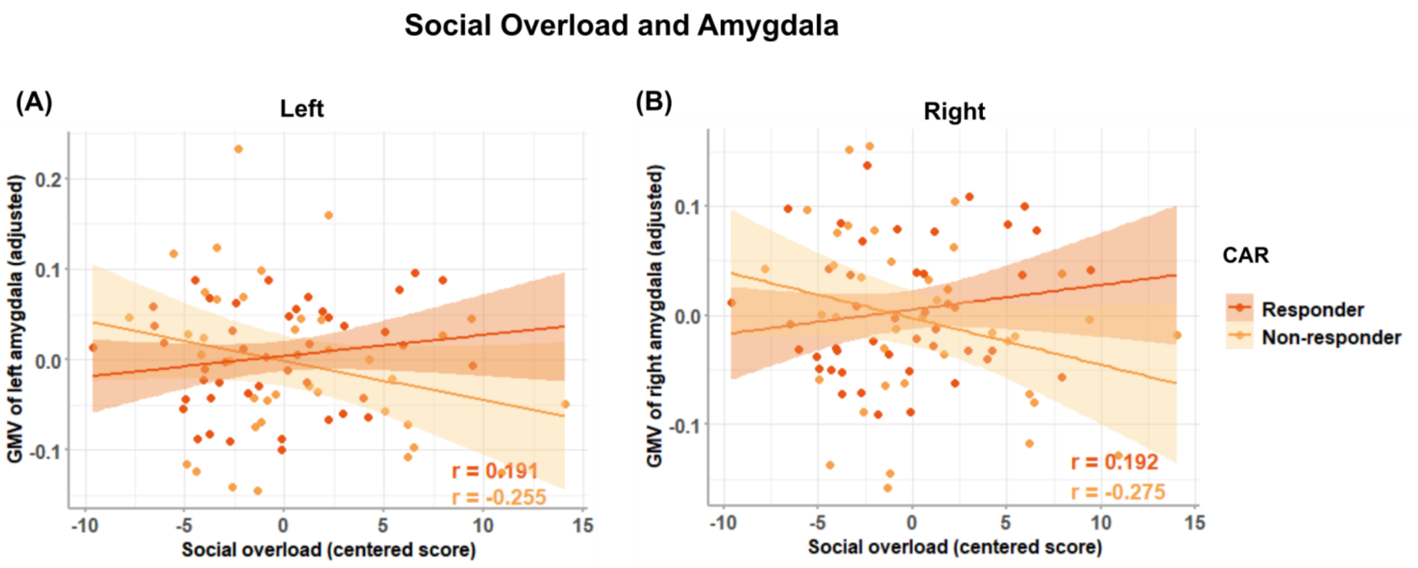

Figure S2. Associations between social overload and GMV of the left (A) and right (B) hippocampus in responders and non-responders. CAR = cortisol awakening response.

Table S5. Statistical parameters for the models on social tension, cortisol awakening response (CAR) and sex and grey matter volume (GMV) of the left and right hippocampus and amygdala.

	**GMV of left hippocampus**		**GMV of right hippocampus**		**GMV of left amygdala**		**GMV of right amygdala**	
	**β**	**t**	**β**	**t**	**β**	**t**	**β**	**t**
Age	0.05	0.58	0.01	0.15	0.10	1.31	0.07	0.85
TIV	0.54	**4.55***	0.63	**5.44***	0.56	**5.09***	0.59	**5.44***
Sex	-0.12	-0.84	-0.05	-0.31	-0.18	-1.30	-0.17	-1.24
CAR	-0.05	-0.44	-0.03	-0.27	-0.02	-0.17	-0.06	-0.53
Social tension	-0.20	-1.50	-0.20	-1.50	-0.16	-1.32	-0.09	-0.73
Social tension × sex	0.29	**2.36***	0.26	**2.14***	0.18	1.59	0.18	1.60
Social tension × CAR	-0.27	**-2.16***	-0.24	**-1.99***	-0.14	-1.22	-0.13	-1.13
Sex × CAR	0.04	0.31	0.01	0.10	-0.04	-0.32	-0.01	-0.05
	R^2 = 0.515, p < 0.001		R^2 = 0.552, p < 0.001		R^2 = 0.589, p < 0.001		R^2 = 0.591, p < 0.001	

Note: TIV = total intracranial volume. * indicate significant at $p < 0.05$ uncorrected.

Table S6. Statistical parameters for the models on social overload, CAR and sex and GMV of the left and right hippocampus and amygdala.

	**GMV of left hippocampus**		**GMV of right hippocampus**		**GMV of left amygdala**		**GMV of right amygdala**	
	**β**	**t**	**β**	**t**	**β**	**t**	**β**	**t**

Age	0.06	0.67	0.02	0.24	0.11	1.38	0.09	1.14
TIV	0.63	**5.06***	0.71	**5.87***	0.62	**5,70***	0.66	**6.31***
Sex	-0.03	-0.22	0.04	0.25	-0.10	-0.77	-0.10	-0.73
CAR	0.01	0.05	0.02	0.19	0.03	0.26	-0.01	-0.09
Social overload	0.06	0.39	0.04	0.31	0.12	0.96	0.10	0.78
Social overload × sex	0.08	0.69	0.08	0.70	0.03	0.27	0.09	0.92
Social overload × CAR	-0.23	-1.76	-0.21	-1.65	-0.25	**-2.19***	-0.28	**-2.50***
Sex × CAR	-0.02	-0.22	-0.05	-0.35	-0.09	-0.71	-0.05	-0.39
	R^2 = 0.456, p < 0.001		R^2 = 0.049, p < 0.001		R^2 = 0.582, p < 0.001		R^2 = 0.609, p < 0.001	

Note: TIV = total intracranial volume. * indicate significant at p < 0.05 uncorrected.

Table S7. Statistical parameters for the models on lack of social recognition, CAR and sex and GMV of the left and right hippocampus and amygdala.

	**GMV of left hippocampus**		**GMV of right hippocampus**		**GMV of left amygdala**		**GMV of right amygdala**	
	**β**	**t**	**β**	**t**	**β**	**t**	**β**	**t**
Age	0.03	0.38	-0.01	-0.07	0.08	1.07	0.06	0.79
TIV	0.60	**4.84***	0.69	**5.72***	0.61	**5.64***	0.65	**6.03***
Sex	-0.07	-0.42	0.01	0.07	-0.13	-0.93	-0.13	-0.97
CAR	0.02	0.12	0.03	0.25	0.05	0.45	-0.01	-0.12
Lack of social recognition	-0.08	-0.56	-0.03	-0.20	-0.03	-0.24	0.02	0.19

Lack of social recognition × sex	0.15	1.26	0.11	0.95	0.10	0.99	0.10	0.92
Lack of social recognition × CAR	-0.13	-1.05	-0.15	-1.28	-0.20	-1.87	-0.18	-1.67
Sex × CAR	-0.02	-0.13	-0.05	-0.32	-0.10	-0.78	-0.03	-0.26
	$R^2 = 0.451$, $p < 0.001$		$R^2 = 0.486$, $p < 0.001$		$R^2 = 0.587$, $p < 0.001$		$R^2 = 0.592$, $p < 0.001$	

Note: TIV = total intracranial volume. * indicate significant at p < 0.05 uncorrected.

Table S8. Statistical parameters for the models on social isolation, CAR and sex and GMV of the left and right hippocampus and amygdala.

	**GMV of left hippocampus**		**GMV of right hippocampus**		**GMV of left amygdala**		**GMV of right amygdala**	
	**β**	**t**	**β**	**t**	**β**	**t**	**β**	**t**
Age	0.05	0.57	0.01	0.09	0.10	1.24	0.08	1.00
TIV	0.60	**4.78***	0.68	**5.58***	0.60	**5.44***	0.63	**5.85***
Sex	-0.08	-0.48	0.00	0.00	-0.13	-0.97	-0.13	-0.99
CAR	-0.04	-0.34	-0.01	-0.12	-0.01	-0.12	-0.05	-0.50
Social isolation	-0.01	-0.06	-0.01	-0.05	0.08	0.64	-0.03	-0.22
Social isolation × sex	-0.05	-0.44	-0.02	-0.18	-0.05	-0.45	0.01	0.14
Social isolation × CAR	0.12	1.01	0.05	0.41	0.07	0.67	0.07	0.71
Sex × CAR	0.04	0.25	-0.01	-0.06	-0.05	-0.34	0.01	0.05
	$R^2 = 0.437$, $p < 0.001$		$R^2 = 0.467$, $p < 0.001$		$R^2 = 0.564$, $p < 0.001$		$R^2 = 0.574$, $p < 0.001$	

Note: TIV = total intracranial volume. * indicate significant at p < 0.05 uncorrected.
